# Comparison of stresses in monoblock tilted implants and conventional angled multiunit abutment-implant connection systems in the all-on-four procedure

**DOI:** 10.1186/s12903-021-02023-y

**Published:** 2021-12-16

**Authors:** Özge Özdal Zincir, Ateş Parlar

**Affiliations:** 1 Department of Oral and Maxillofacial Surgery, Faculty of Dentistry, Istanbul Gelisim University, Istanbul, Turkey; 2 Department of Periodontology, Private Primadent Oral, Dental Health and Implantology Center, Ankara, Turkey

**Keywords:** All-on-four, Finite element analysis, Fixed prosthesis, Monoblock implant, Tilted implant

## Abstract

**Background:**

The All-on-four dental implant method is an implantology method designed to provide a comfortable prosthetic treatment option by avoiding advanced surgical procedures. This research aims to compare and evaluate the stress and tension values in conventional angled multiunit abutment-implant connection systems and monoblock dental implants used in the all-on-four procedure with finite element analysis.

**Methods:**

Two master models were created by placing four implants connected to multiunit abutments (group A) in the interforaminal region of a completely edentulous mandible and four monoblock implants (group B) in the same region of another completely edentulous mandible. Group A implants were classified according to their diameter as follows: 3.5 mm (M1A), 4.0 mm (M2A), and 4.5 mm (M3A). Similarly, group B implants were classified as M1B, M2B, and M3B. In the six models rehabilitated with acrylic fixed prostheses, a 100 N force was applied to the anterior implant region, and a 250 N force was applied to the posterior cantilever in both axial and 30° oblique directions. Von Mises stresses were analyzed in the bone and implant regions of all models.

**Results:**

M1A and M1B, M2A and M2B, and M3A and M3B were compared with each other under axial and oblique forces. The maximum Von Mises stresses in the bone around implants and the prosthesis screws, and the maximum and minimum principal stresses in the cortical and trabecular bone in group A models were significantly higher than those in group B models.

**Conclusions:**

In monoblock implant systems under axial and oblique forces, higher stress is accumulated in the bone, prosthesis screw and implant compared to multiunit abutment-implant connection systems.

## Background

Dental implant-supported restorations are preferred over complete dentures as they provide edentulous patients with a more comfortable treatment option. Moreover, they can counter problems such as retention failure, lack of stability, and patient dissatisfaction associated with complete dentures [1]. However, there are some limiting factors in dental implant surgery, especially in edentulous patients. These factors include poor bone quality in the posterior jaw region, decreased alveolar bone volume, and enlargement of the lower wall of the maxillary sinus towards the alveolar ridge [2, 3]. Advanced surgical practices and graft materials are required to eliminate these limitations, thus increasing the duration and cost of treatment [4].

Eliasson et al. [5] suggested that four implants can be distributed between the mental foramina to receive fixed restorations in edentulous patients. However, a limited bone volume between the mental foramina necessitates the fabrication of prostheses with long-span cantilevered segments distal to posterior implants. Moreover, the presence of cantilevers in fixed restorations can augment the load on implants up to two or three times due to bending moments. Some alternatives have been proposed to overcome these limitations, such as placing a short implant distal to the mental foramina and combining the cantilever segment with these implants [6].

Malo et al. [7] developed the All-on-four treatment protocol (Nobel BioCare AG, Kloten, Switzerland) based on the immediate loading of four dental implants placed between the mental foramina. In the All-on-four system, two anterior implants are placed straight and parallel to each other, and two posterior implants are tilted distally to a maximum of 45°. This procedure decreases the cantilever length and the risk of stress accumulation and bone resorption at the implant-bone interface [7].

Angled multiunit abutments are used for tilted distal implants in the traditional All-on-four procedure. Microorganisms and other toxic substances can accumulate in the microspaces between the multiunit abutment and the implant body, increasing the risk of peri-implantitis [8, 9]. Moreover, screw fractures that result from abutment screw loosening are among multiunit abutment complications [10].

Monoblock tilted dental implant system is a one-piece system that does not have any components such as abutment screws between the implant and abutment. It has been used as an alternate to the multiunit abutment-implant connection system in the All-on-four procedure recently.

In the biomechanical analysis of dental implants, the finite elemet method (FEM) is frequently used because it reflects the complexity of clinical conditions and has benefits over many analysis methods [11]. The purpose of this study is to compare the stress and strain values of the monoblock tilted dental implant system with the conventional angled multiunit abutment-implant connection system in the implant parts and surrounding bone using FEM.

## Methods

**Fig. 1:**
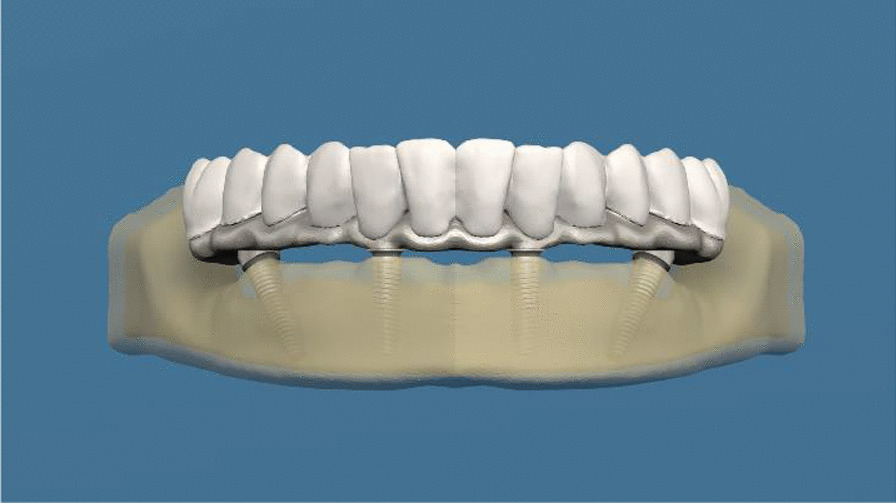
Finite element model of all-on-four procedure with implants of the multiunit abutment-implant connection system

**Fig. 2:**
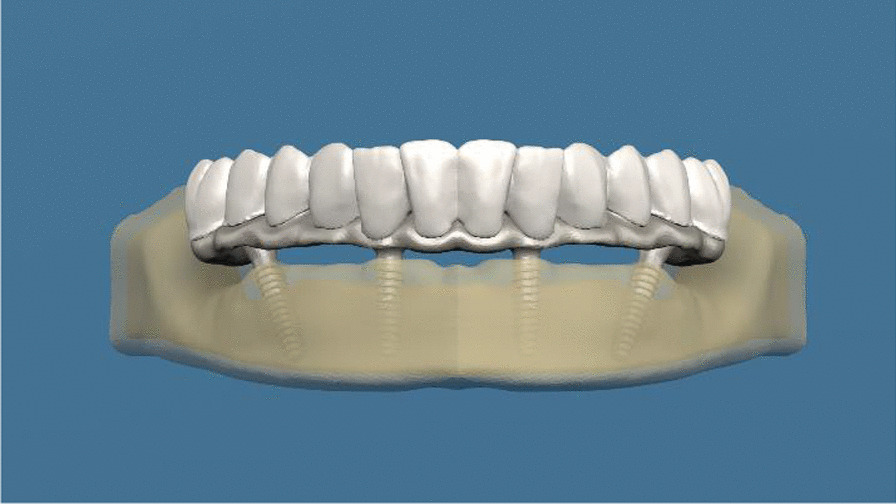
Finite element model of all-on-four procedure with implants of the monoblock implant system

Four implants of the multiunit abutment-implant connection system (Oxy Implant by Biomec S.r.l, Colico, Italy) [group A] and four monoblock implants (Oxy Implant by Biomec S.r.l, Colico, Italy) [group B] were installed in the interforaminal area of the edentulous mandible, and two different 3-D finite element models were prepared and rehabilitated with an acrylic fixed prosthesis. The anterior implants used in the models were placed straight, and the posterior implants at 30° angles (Figs. 1 and 2). A total of 6 models were obtained from each system using implants with 3.5 mm, 4.0 mm, and 4.5 mm diameters.

Dental volumetric tomography of the edentulous mandible was used to get finite element models. To optimize the 3-dimension (3-D) network structure and make it more homogeneous, generate the 3-D solid model, and the FEM analysis; Intel Xeon ® R CPU 3.30 GHz processor, 500 GB Hard disk, a computer equipped with 14 GB RAM and Windows 7 Ultimate Version Service Pack 1 operating system, Activity 880 (smart optics Sensortechnik GmbH, Bochum, Germany), an optical scanner and 3-D scanner, Rhinoceros 4.0 (Seattle, WA 98103 USA), 3-D modeling software, VRMesh Studio (VirtualGrid Inc, Bellevue City, WA, USA), and Algor Fempro (ALGOR, Inc., PA 152382932 USA) analysis program were used to model cortical bone, trabecular bone, body of implant, abutment, and prosthetic materials.

All the materials used in the program and models were considered linear elastic, homogeneous, and isotropic. As the biological properties of the materials used in the models do not have accepted universal values, the average values were obtained from the literature (Table 1).

**Table 1:** Properties of materials in models

	Modulus of elasticity (GPa)	Poisson’s ratio
Acrylic resin (prosthesis)	2.7	0.35
Cortical bone	13.0	0.3
Trabecular bone	5.5	0.3
Titanium implant and abutment (Grade 4 titanium)	102.0	0.35
Titanium substructure in prosthesis (Ti–6Al–4V)	110.0	0.28

### Bone

The edentulous mandible bone was modeled with a height of 15 mm, a thickness of 7 mm, and an interforamina range of 46 mm. The cortical bone heights in the upper and lower layers of the model were 3 mm and 2 mm, respectively. The trabecular bone height modeled between the two cortical layers was 10 mm.

### Dental implants and abutments

In the multiunit abutment-implant connection system groups, conical connection implants with 3.5 mm, 4.0 mm, 4.5 mm diameters, and 11.5 mm length were used in the anterior area. The same diameter implants with 13 mm length were used in the posterior region. Also, 30° angulated multiunit abutments were used for tilted posterior implants, and straight multiunit abutments were used for anterior implants.

In the monoblock implant system groups, straight monoblock implants with 3.5 mm, 4.0 mm, 4.5 mm diameters, and 11.5 mm length were used in the anterior region. Monoblock implants with the same implant diameters, 13 mm length, and a 30° tilt were used in the posterior area.

Implants in the anterior area were installed as far as possible from each other, with a confident distance of 12 mm between implants in the posterior region.

### Prosthesis

A minimum total prosthesis thickness of 1.5 mm is suggested for resistance to fracture [12]. In this study, a 2.2 mm thick homogeneous acrylic resin block with a 10 mm cantilever on both ends and a titanium substructure was created.

### Loading procedure

**Fig. 3:**
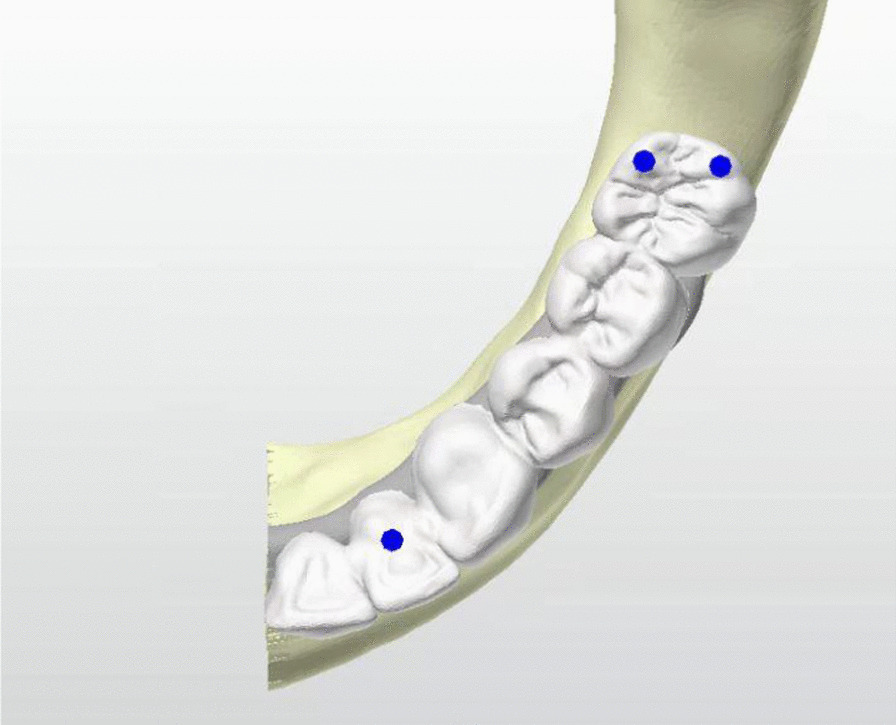
Force applied zones in models

A 100 N force was carried out to the anterior implants, and a 250 N force was carried out to the mesiobuccal and distobuccal ends of the cantilever in the posterior region (Fig. 3). Both axial and oblique forces angled at 30° to the long axis were applied to the force zones in each model.

### Meshed models

**Fig. 4:**
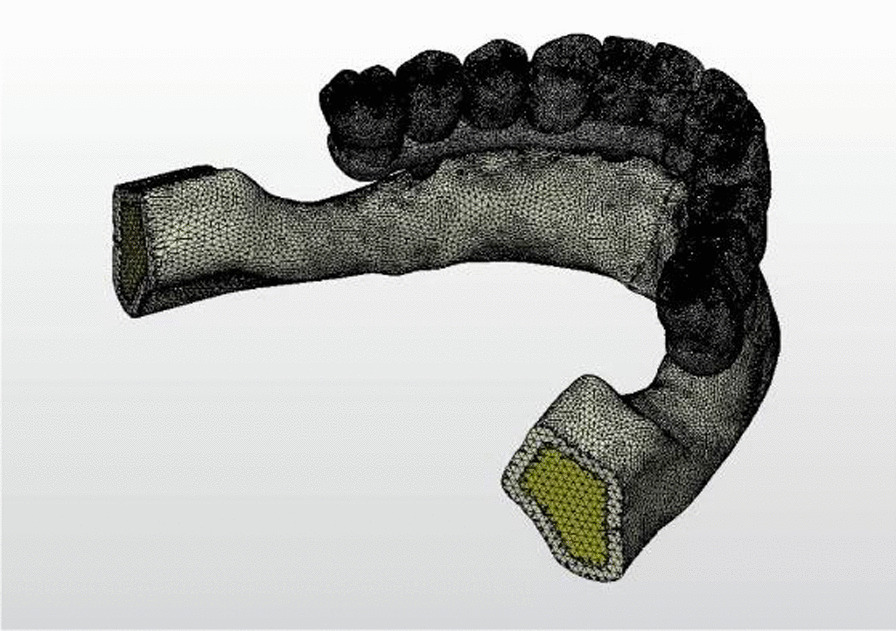
Meshed model

These finite element analysis models were transferred to Algor Fempro (Algor Inc., USA) software in STL format for analysis. They were created geometrically using VRMesh software for meshing (Fig. 4).

In the meshing process, models were made of 10 node (brick type) elements as far as possible. In the regions close to the center, fewer nodes were used to complete the structure when necessary. The models were converted into solid bricks and tetrahedral elements. In bricks and tetrahedral modeling, Fempro uses 8-noded elements as much as it can and 7-node, 6-node, 5-node, and 4-node elements where 8-node elements cannot reach the required details. To obtain realistic results, considering the dimensions of the mandible bone model, we selected as many elements as possible. A total of six mathematical models were created with implants of different diameters and categorized into two main groups: the multiunit abutment-implant connection implants group (group A) and the monoblock implants group (group B). Group A implants were classified according to their diameter as follows: 3.5 mm (model-1A [M1A]), 4.0 mm (model-2A [M2A]), and 4.5 mm (model-3A [M3A]). Group B implants were classified as follows: 3.5 mm (model-1B [M1B]), 4.0 mm (model-2B [M2B]), and 4.5 mm (model-3B [M3B]). The number of elements and knots used in the created mathematical models are presented in Table 2.

**Table 2:** Number of nodes and number of elements in models

Models in groups	Number of nodes	Number of elements
M1A	281,407	1,329,234
M2A	261,731	1,286,671
M3A	266,199	1,313,308
M1B	320,877	1,317,592
M2B	314,863	1,357,176
M3B	320,458	1,396,542

### Comparative groups

The stress rates in the implants and prosthesis screws of the same diameter in the same area were compared to the stress rates in the bone under axial and oblique loading. The determined forces were applied to the force regions of the six models concurrently.

### Measurements of stress and strain values

In loadings using the FEM program, von Mises standard were used to assess the tension in the implants and prosthesis screws, and maximum principal stresses were used to evaluate the tension in the cortical and trabecular bone.

## Results

The maximum von Mises stress (VMS) (MPa) values in the implants and prosthesis screws and the minimum–maximum principal stress (PS) (MPa) values in the cortical and trabecular bone are shown in Tables 3, 4, 5 and 6, and their distribution is shown in Figs. 5, 6, 7, 8, 9, 10, 11, 12, 13, 14, 15 and 16.

**Table 3:** Maximum VMS around implants (MPa)

Model	Axial force		Oblique force	
	Anterior implant	Posterior implant	Anterior implant	Posterior implant
M1A	64.78	143.06	255.51	555.95
M2A	57.04	149.48	208.10	505.82
M3A	70.37	177.96	240.35	424.52
M1B	275.08	429.99	752.01	1025.61
M2B	210.86	359.12	652.88	795.31
M3B	181.36	320.53	426.72	703.75

**Table 4:** Maximum VMS in prosthesis screws of implants (MPa)

Model	Axial force		Oblique force	
	Anterior implant’s screw	Posterior implant’s screw	Anterior implant’s screw	Posterior implant’s screw
M1A	16.97	58.81	40.12	96.47
M2A	16.70	61.50	39.76	55.06
M3A	16.39	57.74	38.56	56.05
M1B	113.23	345.10	342.16	800.37
M2B	190.81	316.80	596.84	723.70
M3B	122.13	345.82	389.60	827.17

**Table 5:** Maximum–minimum PS in cortical bone (MPa)

Model	Axial force				Oblique force			
	Maximum PS cortical bone around the anterior implant	Minimum PS cortical bone around the anterior implant	Maximum PS cortical bone around the posterior implant	Minimum PS cortical bone around the posterior implant	Maximum PS cortical bone around the anterior implant	Minimum PS cortical bone around the anterior implant	Maximum PS cortical bone around the posterior implant	Minimum PS cortical bone around the posterior implant
M1A	12.99	21.11	9.97	43.62	34.12	33.52	64.75	96.63
M2A	15.71	20.58	13.41	47.55	28.21	33.50	66.15	104.18
M3A	13.72	18.54	9.79	44.78	17.43	27.40	48.17	90.95
M1B	34.25	40.39	25.02	155.56	115.32	125.06	154.02	277.28
M2B	36.80	46.62	23.27	110.79	112.66	93.94	123.58	242.73
M3B	27.28	28.63	19.08	89.07	67.15	82.76	99.72	190.66

**Table 6:** Maximum–minimum PS in trabecular bone (MPa)

Model	Axial force				Oblique force			
	Maximum PS trabecular bone around the anterior implant	Minimum PS trabecular bone around the anterior implant	Maximum PS trabecular bone around the posterior implant	Minimum PS trabecular bone around the posterior implant	Maximum PS trabecular bone around the anterior implant	Minimum PS trabecular bone around the anterior implant	Maximum PS trabecular bone around the posterior implant	Minimum PS trabecular bone around the posterior implant
M1A	3.60	4.03	8.79	11.03	9.87	10.01	19.19	23.58
M2A	3.35	5.16	7.15	16.10	6.89	10.30	17.19	24.49
M3A	3.39	3.64	8.95	17.63	5.74	13.52	18.27	27.67
M1B	8.44	9.31	6.71	9.90	21.58	19.20	14.00	17.04
M2B	5.07	5.02	7.09	10.49	10.12	17.97	14.34	22.23
M3B	12.13	5.38	25.08	12.41	8.87	12.98	23.82	37.43

**Fig. 5:**
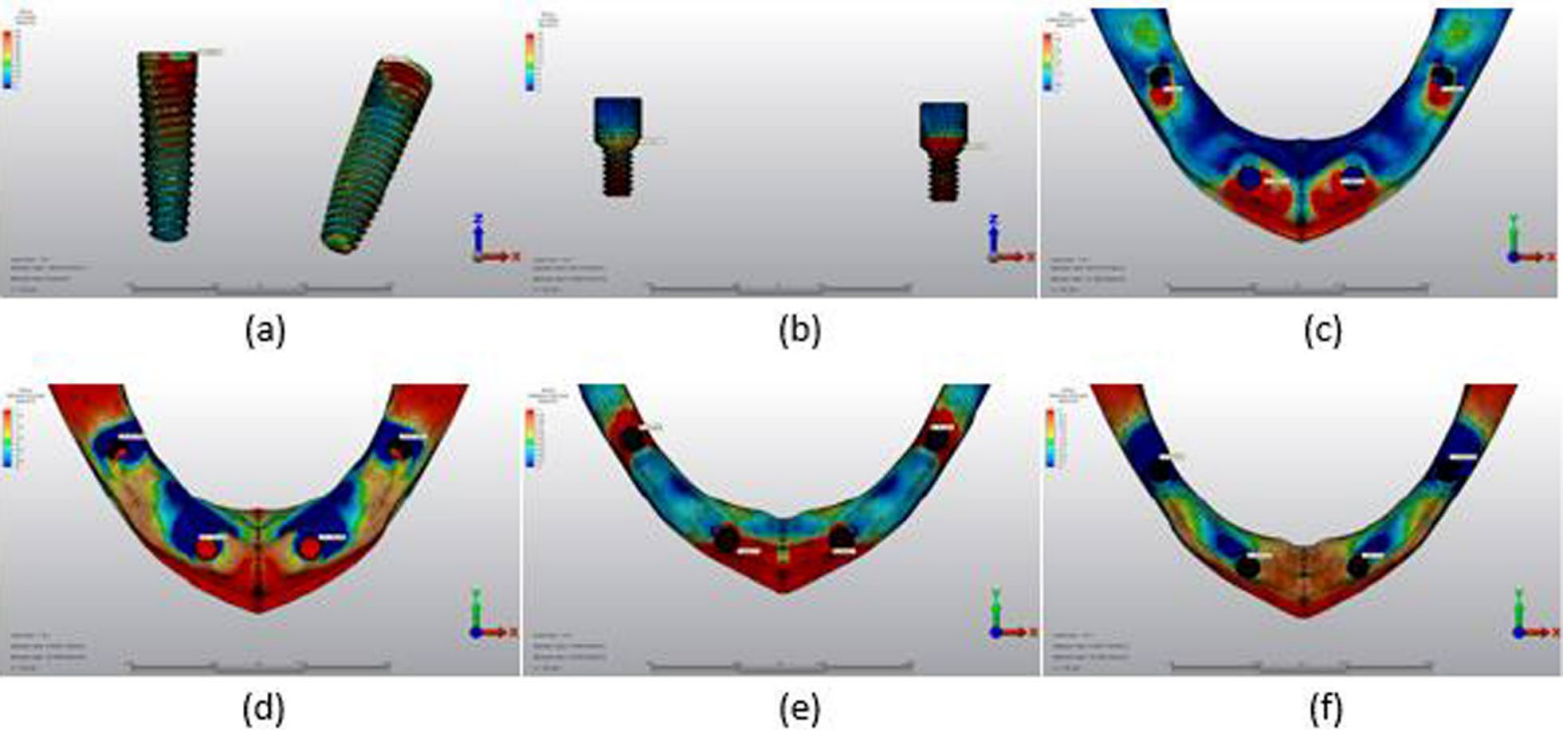
Axial loading in M1A. **a** The maximum VMS around implants; **b** the maximum VMS in prosthesis screws of implants; **c** maximum PS in cortical bone; **d** minimum PS in cortical bone; **e** maximum PS in trabecular bone; **f** minimum PS in trabecular bone

**Fig. 6:**
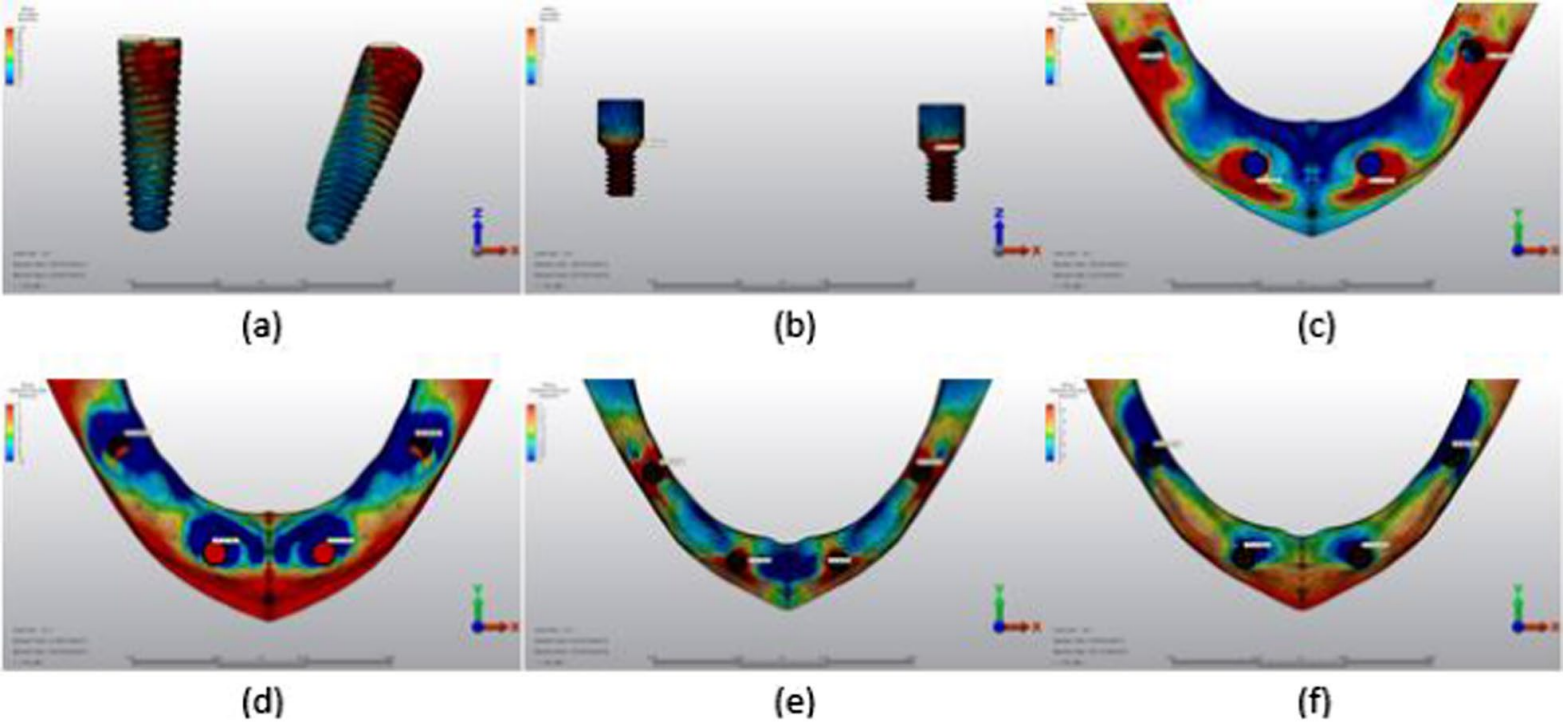
Oblique loading in M1A. **a** The maximum VMS around implants; **b** the maximum VMS in prosthesis screws of implants; **c** maximum PS in cortical bone; **d** minimum PS in cortical bone; **e** maximum PS in trabecular bone; **f** minimum PS in trabecular bone

**Fig. 7:**
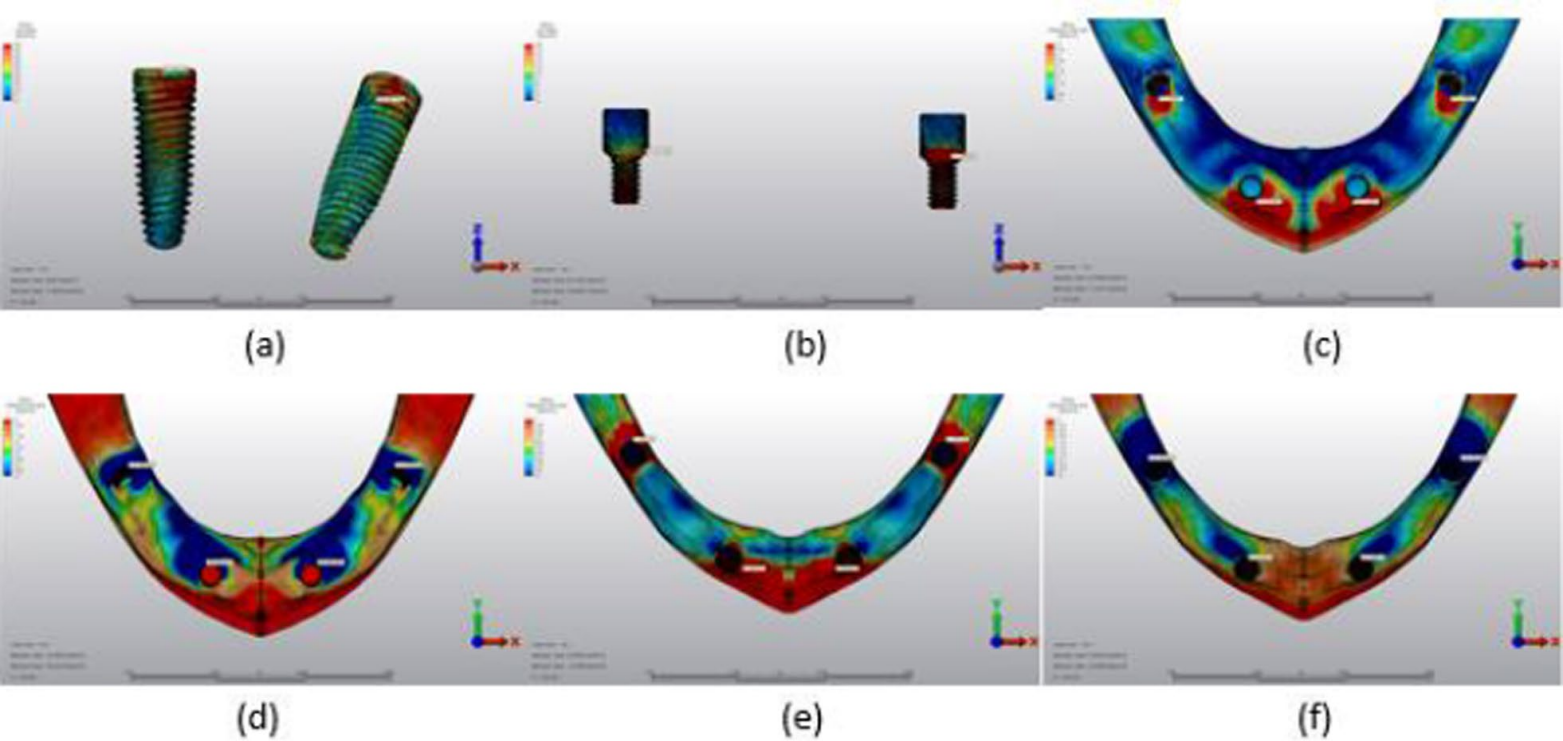
Axial loading in M2A. **a** The maximum VMS around implants; **b** the maximum VMS in prosthesis screws of implants; **c** maximum PS in cortical bone; **d** minimum PS in cortical bone; **e** maximum PS in trabecular bone; **f** minimum PS in trabecular bone

**Fig. 8:**
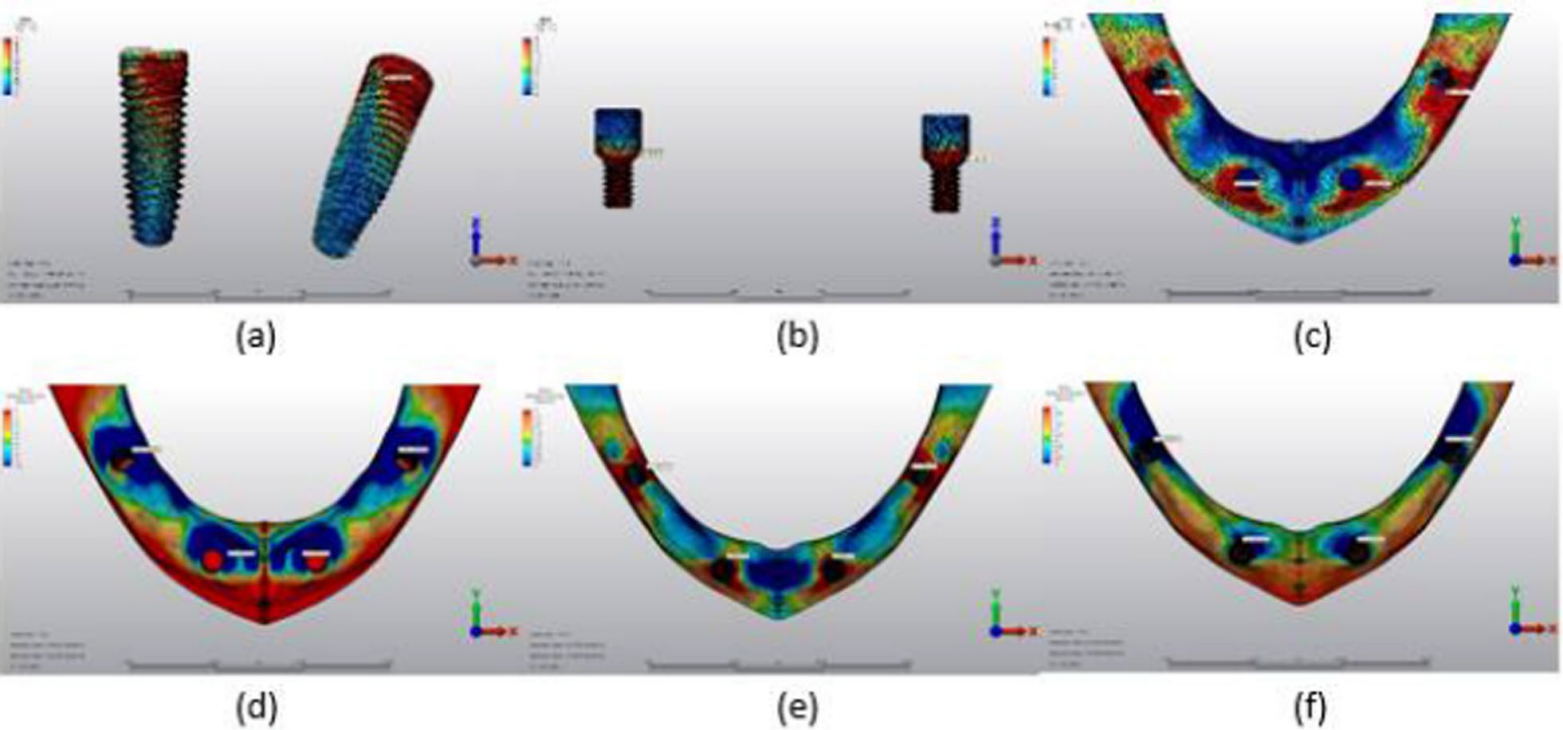
Oblique loading in M2A. **a** The maximum VMS around implants; **b** the maximum VMS in prosthesis screws of implants; **c** maximum PS in cortical bone; **d** minimum PS in cortical bone; **e** maximum PS in trabecular bone; **f** minimum PS in trabecular bone

**Fig. 9:**
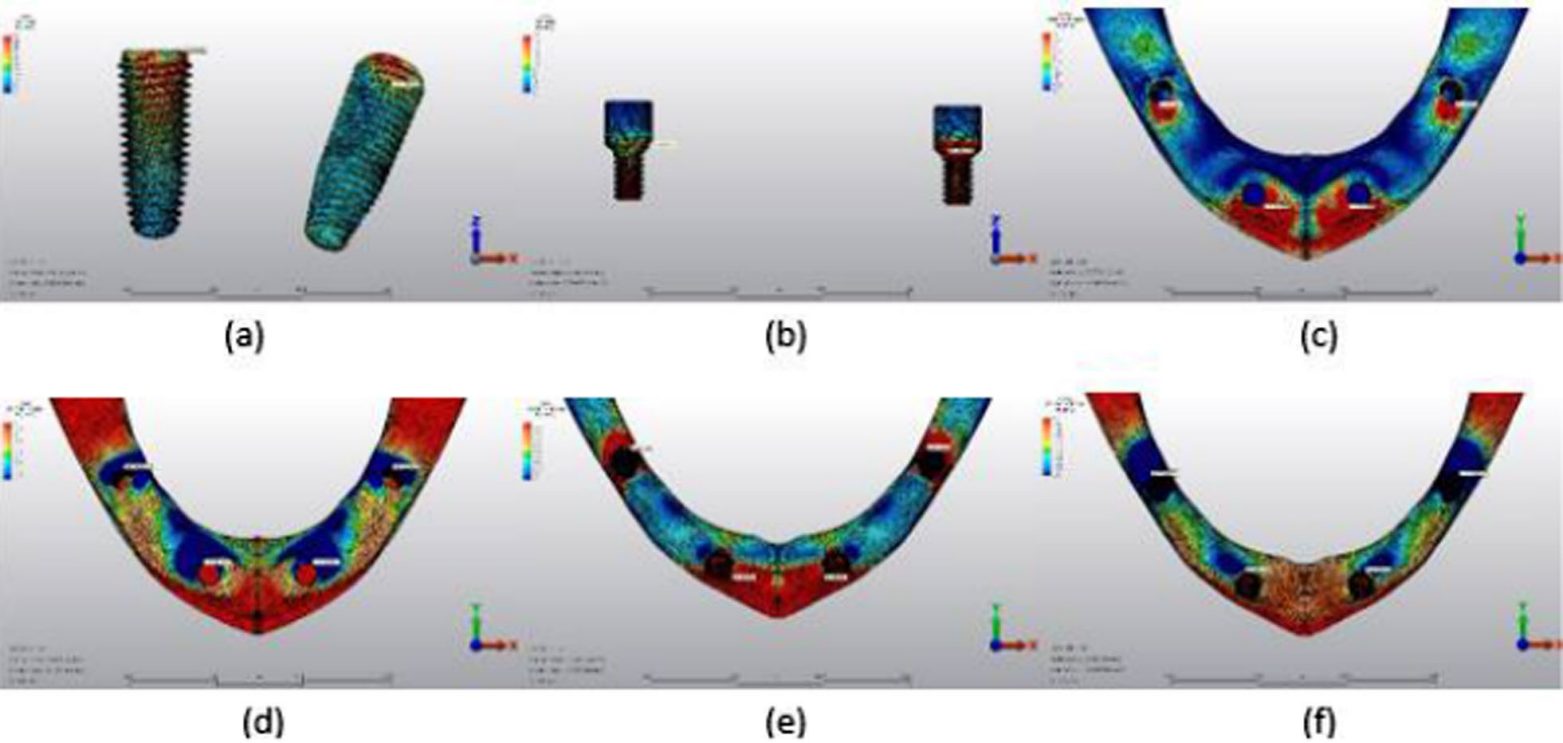
Axial loading in M3A. **a** The maximum VMS around implants; **b** the maximum VMS in prosthesis screws of implants; **c** maximum PS in cortical bone; **d** minimum PS in cortical bone; **e** maximum PS in trabecular bone; **f** minimum PS in trabecular bone

**Fig. 10:**
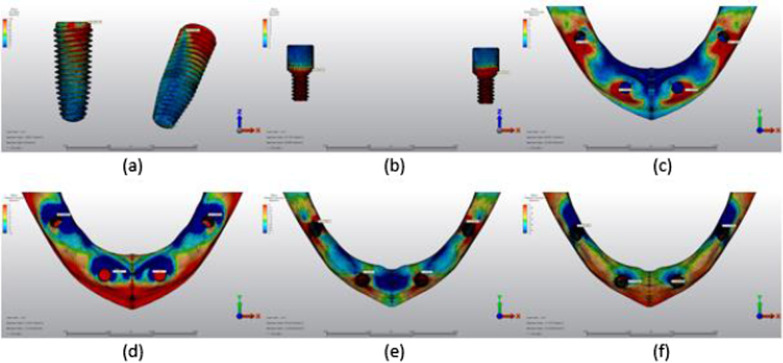
Oblique loading in M3A. **a** The maximum VMS around implants; **b** the maximum VMS in prosthesis screws of implants; **c** maximum PS in cortical bone; **d** minimum PS in cortical bone; **e** maximum PS in trabecular bone; **f** minimum PS in trabecular bone

**Fig. 11:**
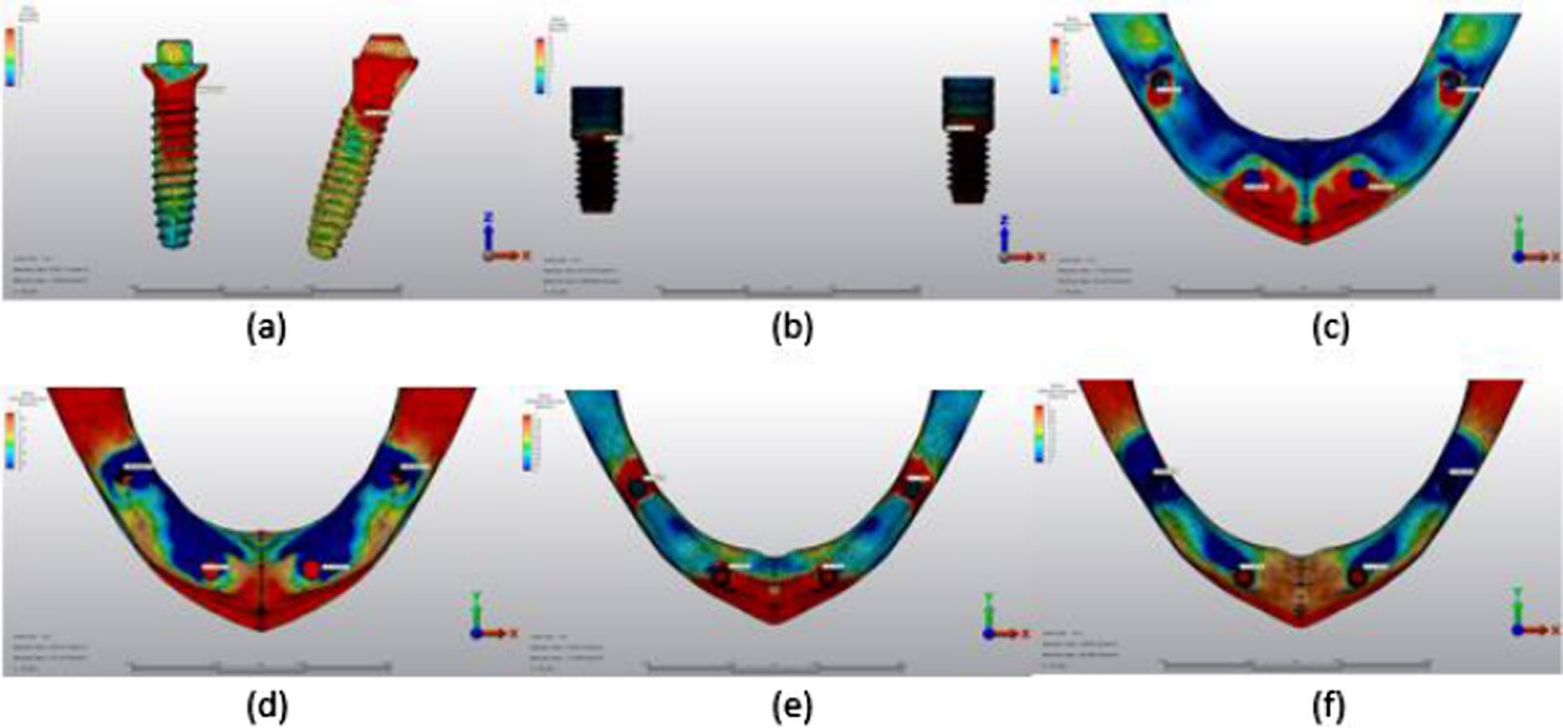
Axial loading in M1B. **a** The maximum VMS around implants; **b** the maximum VMS in prosthesis screws of implants; **c** maximum PS in cortical bone; **d** minimum PS in cortical bone; **e** maximum PS in trabecular bone; **f** minimum PS in trabecular bone

**Fig. 12:**
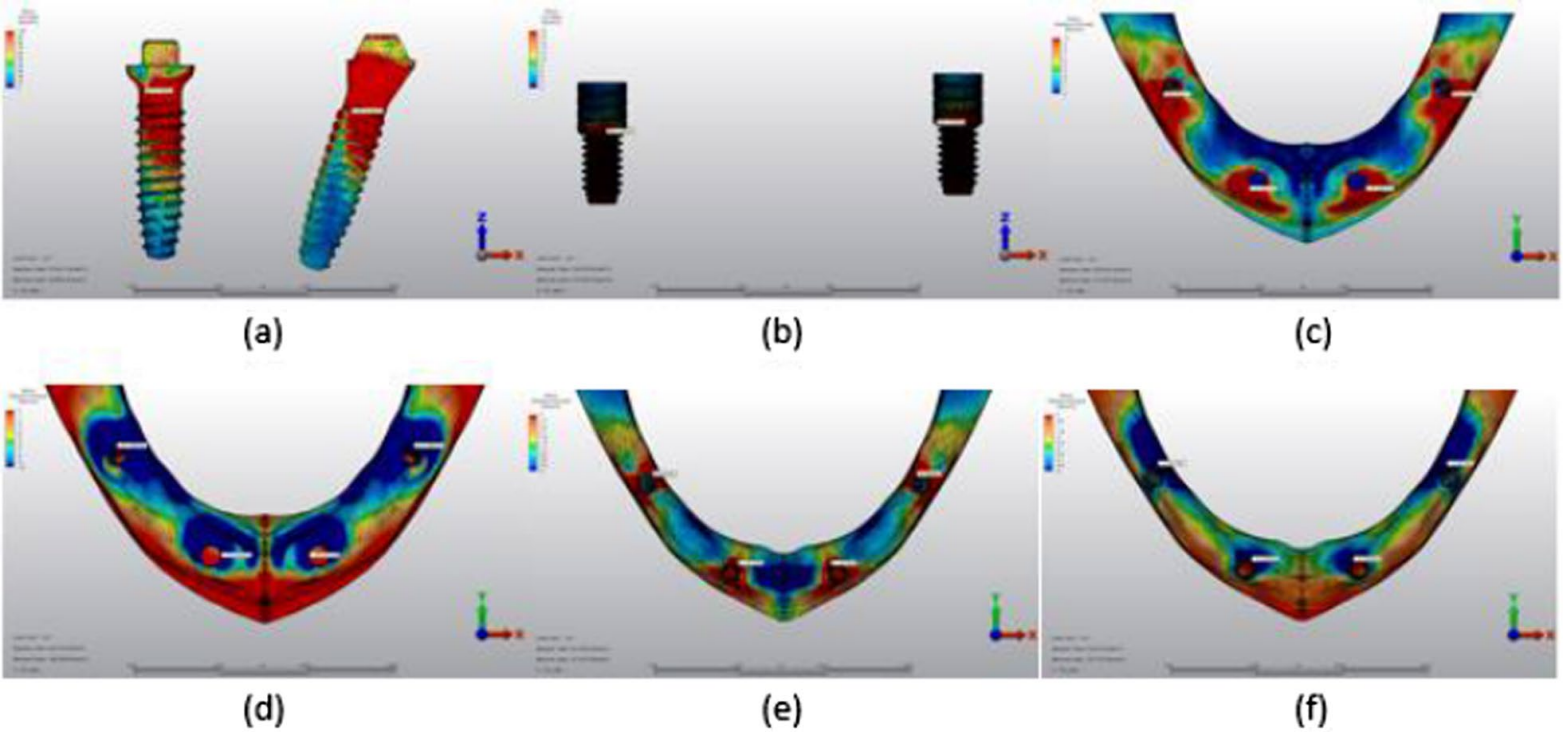
Oblique loading in M1B. **a** The maximum VMS around implants; **b** the maximum VMS in prosthesis screws of implants; **c** maximum PS in cortical bone; **d** minimum PS in cortical bone; **e** maximum PS in trabecular bone; **f** minimum PS in trabecular bone

**Fig. 13:**
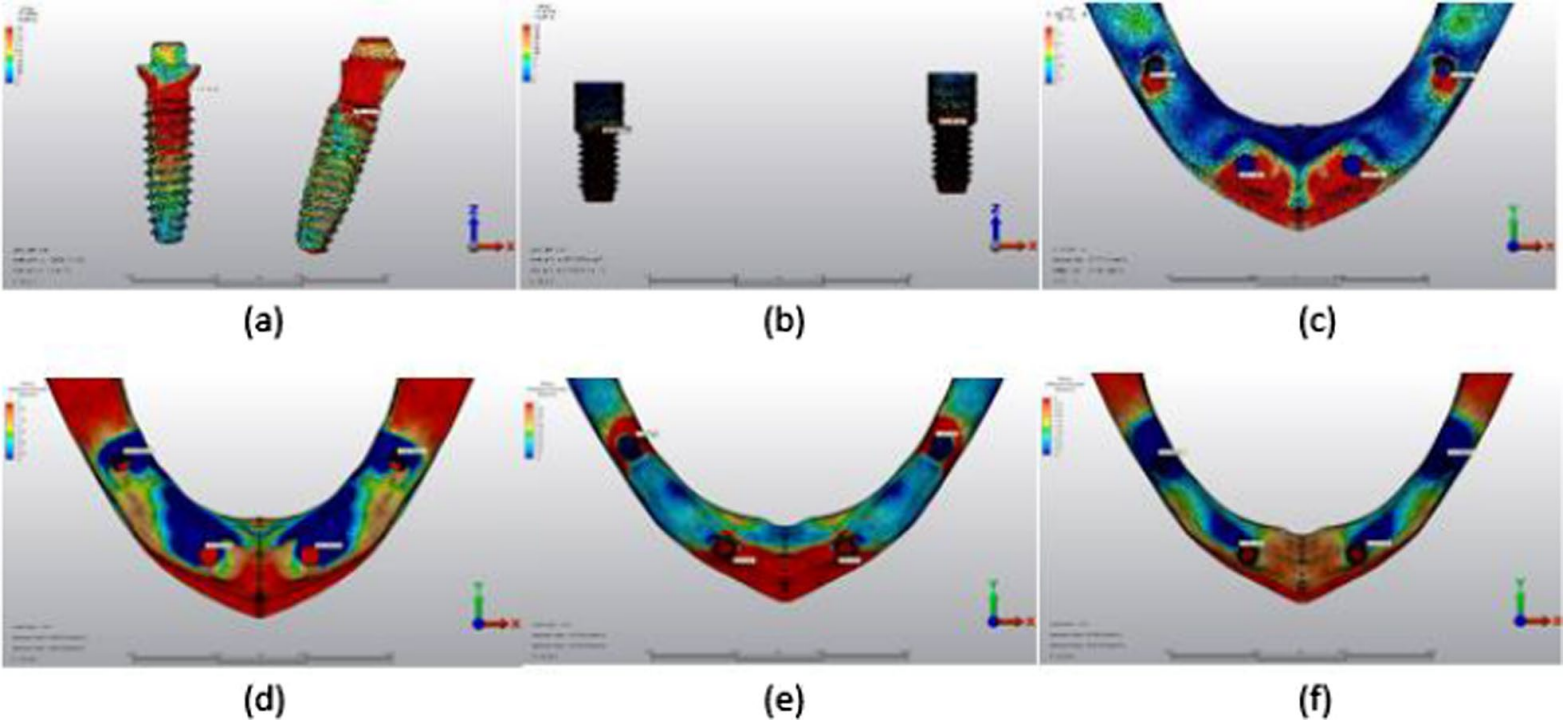
Axial loading in M2B. **a** The maximum VMS around implants; **b** the maximum VMS in prosthesis screws of implants; **c** maximum PS in cortical bone; **d** minimum PS in cortical bone; **e** maximum PS in trabecular bone; **f** minimum PS in trabecular bone

**Fig. 14:**
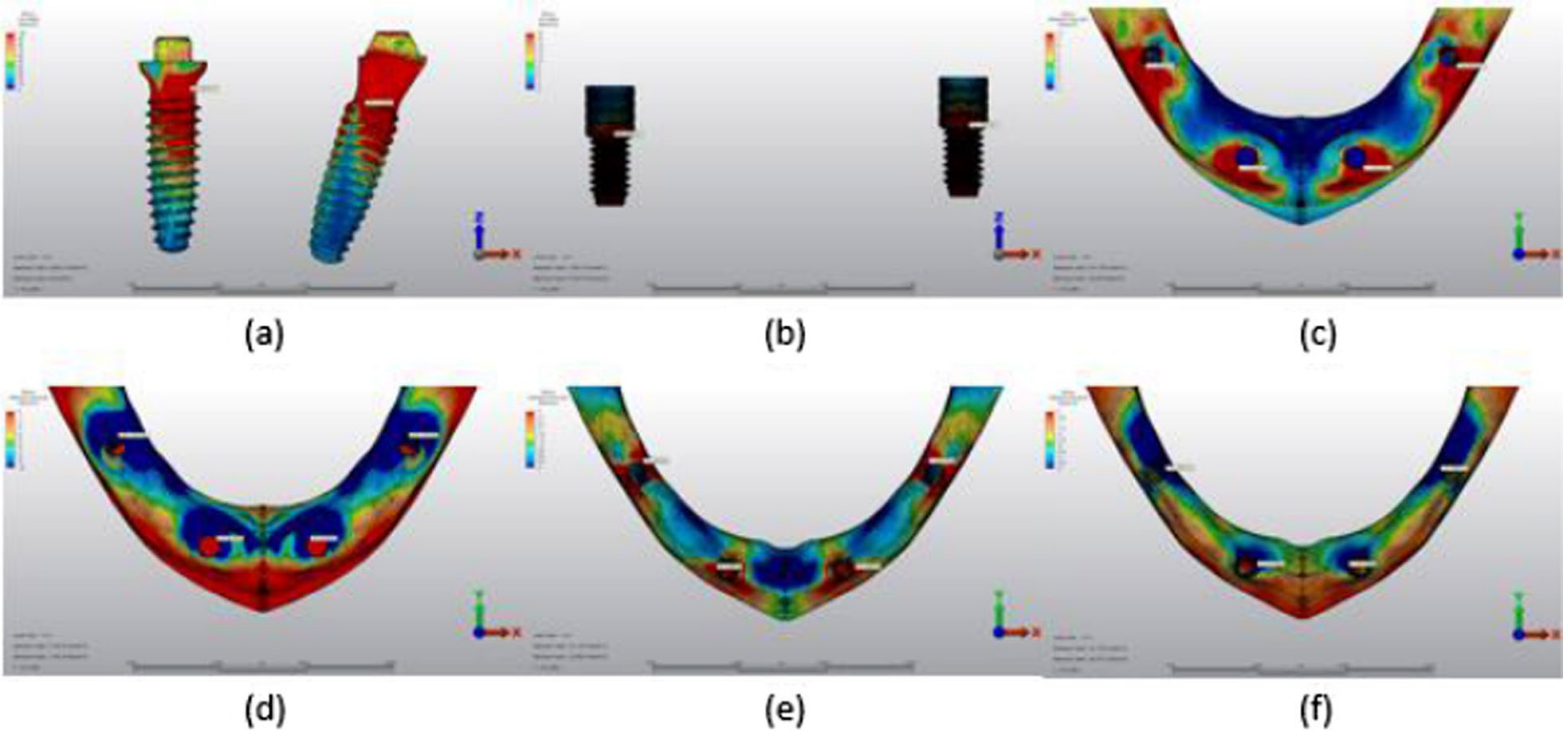
Oblique loading in M2B. **a** The maximum VMS around implants; **b** the maximum VMS in prosthesis screws of implants; **c** maximum PS in cortical bone; **d** minimum PS in cortical bone; **e** maximum PS in trabecular bone; **f** minimum PS in trabecular bone

**Fig. 15:**
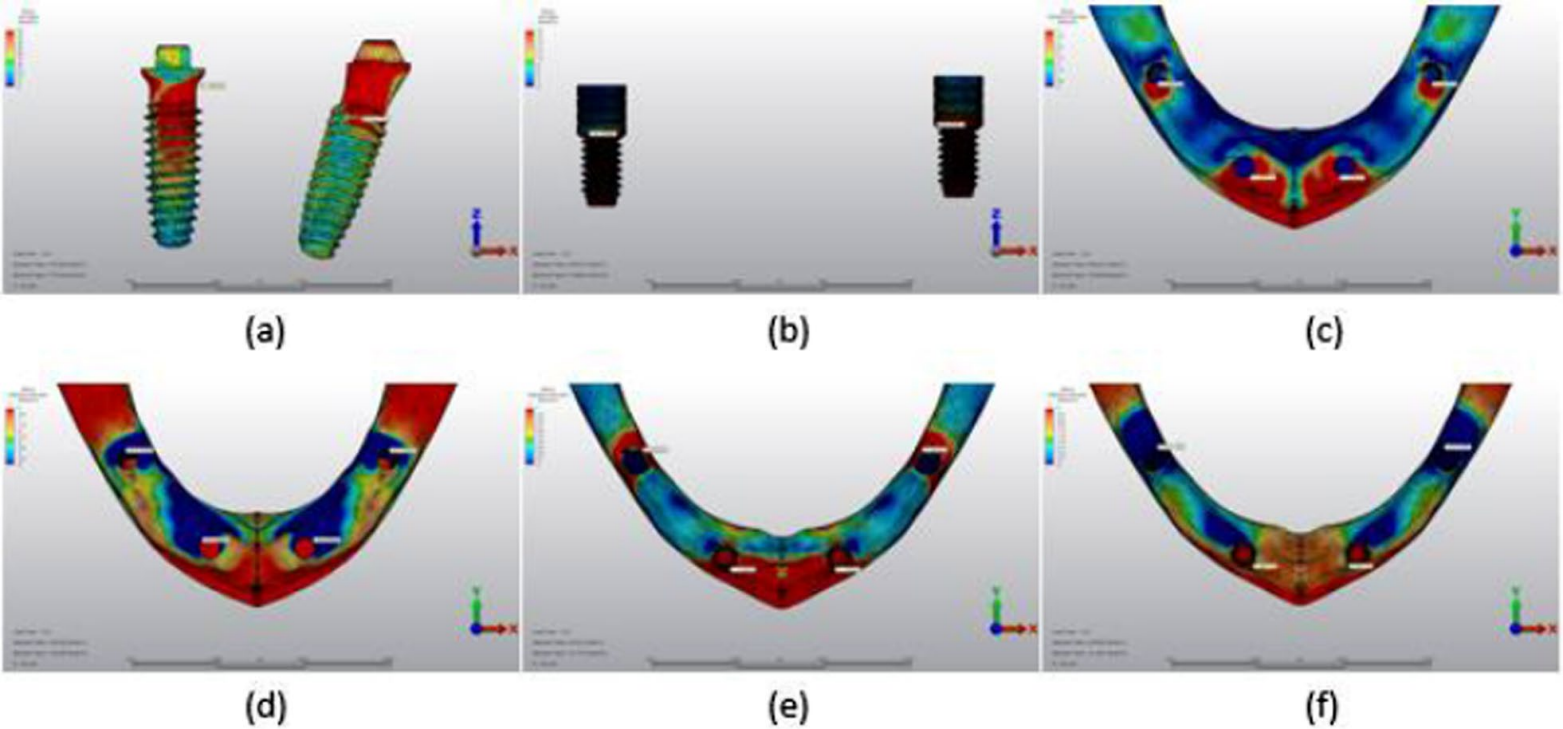
Axial loading in M3B. **a** The maximum VMS around implants; **b** the maximum VMS in prosthesis screws of implants; **c** maximum PS in cortical bone; **d** minimum PS in cortical bone; **e** maximum PS in trabecular bone; **f** minimum PS in trabecular bone

**Fig. 16:**
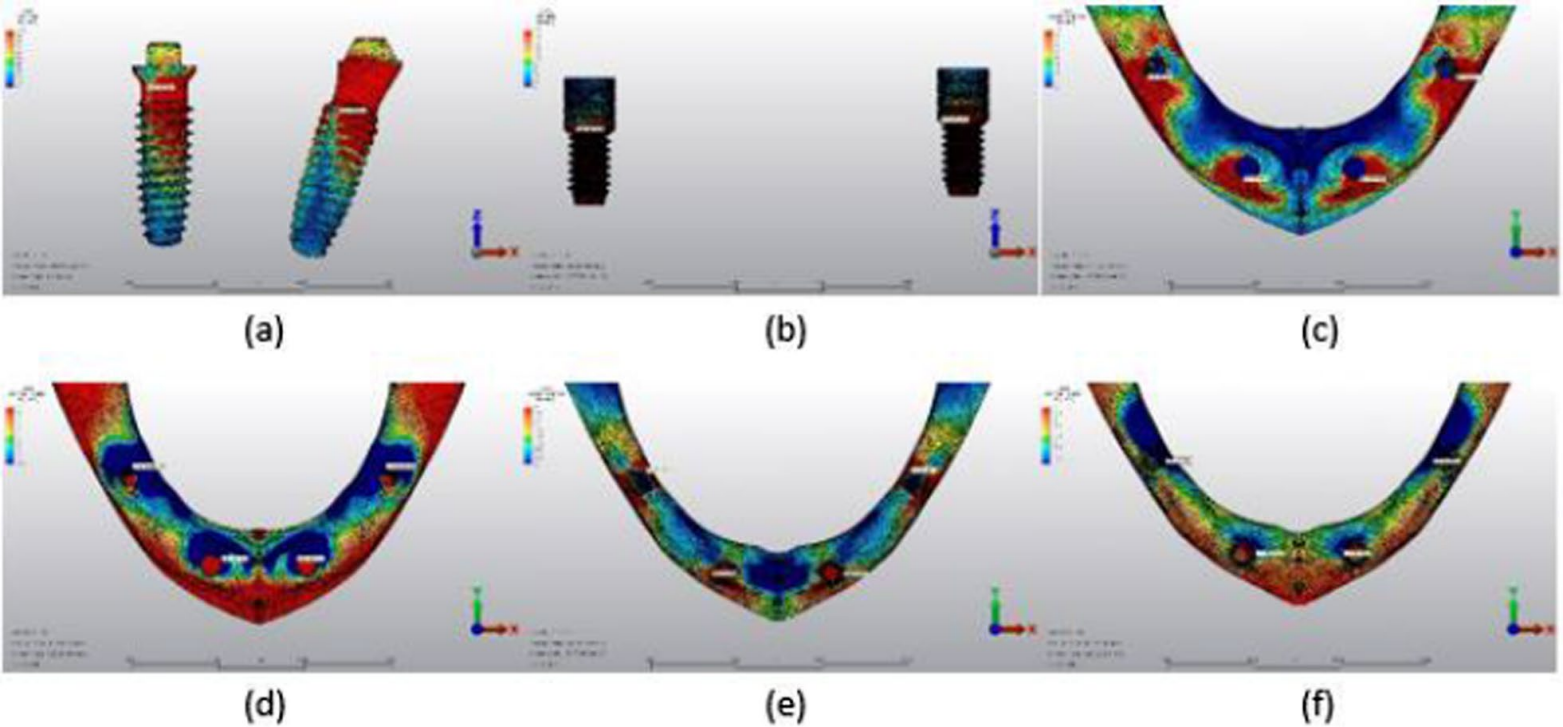
Oblique loading in M3B. **a** The maximum VMS around implants; **b** the maximum VMS in prosthesis screws of implants; **c** maximum PS in cortical bone; **d** minimum PS in cortical bone; **e** maximum PS in trabecular bone; **f** minimum PS in trabecular bone

### Assessment of the models

#### Assessment of VMS in implants

The highest VMS values under axial and oblique loading in both anterior and posterior implants were observed in the M1B model. The lowest VMS values under axial and oblique loading were observed in anterior implants in the M2B model; the lowest VMS values in posterior implants were noted in the M1A model under axial loading and M3A model under oblique loading (Table 3).

#### Assessment of VMS in prosthesis screws of implants

In the anterior implant prosthesis screws of the M2B model and posterior implant prosthesis screws of the M3B model, the highest VMS values were observed under both axial and oblique loading. The lowest VMS values were observed in the M3A model in both anterior and posterior implant prosthesis screws under axial loading; the lowest VMS values under oblique loading were observed in the M3A model in anterior implant prosthesis screws and the M2A model in posterior implant prosthesis screws (Table 4).

#### Assessment of maximum-minimum PS rates in cortical bone

The highest maximum and minimum PS values in the cortical bone around anterior implants were observed in the M2B model, and around posterior implants in the M1B model under axial loading. The highest maximum and minimum PS values in the cortical bone around anterior and posterior implants under oblique loading were also observed in the M1B model.

Under axial loads, the lowest maximum PS in the cortical bone around anterior implants and the lowest minimum PS in the cortical bone around posterior implants were observed in the M1A model. The lowest minimum PS in the cortical bone around anterior implants and the lowest maximum PS in the cortical bone around posterior implants were observed in the M3A model. Under oblique loads, the lowest maximum and minimum PS in the cortical bone around anterior and posterior implants were observed in the M3A model (Table 5).

#### Assessment of maximum-minimum PS rates in trabecular bone

Under axial loads, the highest minimum PS values in the trabecular bone were observed around anterior implants in the M3B model and posterior implants in the M1B model. The highest minimum PS values in the trabecular bone around posterior implants were observed in the M3A model, and the highest maximum PS values in the M3B model. Under oblique loads, the highest maximum and minimum PS in the trabecular bone around anterior implants were observed in the M1B model and posterior implants in the M3B model.

Axial loading of anterior implants revealed the lowest maximum PS in the trabecular bone in the M2A model and the lowest minimum PS in the M3A model. The lowest maximum and minimum PS in the trabecular bone around posterior implants were observed in the M1B model. Under oblique loads, the lowest maximum PS values were observed in the trabecular bone around anterior implants in the M3A model, and the lowest minimum PS values in the M1A model. The lowest maximum and minimum PS in the trabecular bone around posterior implants were observed in the M1B model (Table 6).

## Discussion

Currently, dental implants are a popular treatment option for the rehabilitation of edentulous patients. Moreover, treatment procedures such as the all-on-four concept that use fewer implants with minimally invasive techniques have gained popularity. In monoblock implant applications, the risk of delay in wound healing is high in individuals with systemic disease, heavy smokers and periodontal disease because the implant is not healed off [13]. However, monoblock dental implants considered as a new system in the all-on-four dental implant procedure can prevent peri-implantitis due to microleakage in angled multiunit abutment-implant connection systems and screw loosening due to micro-movements [14, 15]. A systematic review by Mishra et al.[16] reported the presence of microleakage in all multiunit abutments, although the amount of leakage varied according to the torque values applied in the multiunit abutment-implant connection interface with different connection types.

Several studies in the literature suggest that the safe cantilever length is 10 mm when evaluated in terms of stress distribution in fixed prosthesis supported by four implants [17]. Bellini et al.[18] reported that cantilever lengths from 5 to 15 mm lead to increase a 33% stress accumulation at the bone and increase the risk of implant failure. However, Malhotra et al. [19] stated that there was no significant difference between 4 and 12 mm cantilever lengths in this concept. Therefore, we determined the cantilever length as 10 mm in the design of the models in this study.

Dental implant diameter is important when considering stress distribution against chewing forces. Several studies suggest that increased implant diameter reduces stress values in implants [20–22]. These observations differ from the results obtained in some group A models in this study; however, they were consistent with the findings in the B group models. A comparison between both groups to assess stress accumulation in implants showed that stress values in the B group models were higher than those in the A group. The factor in the conflict between the stress values in implants in the A group with the literature may be a limitation of the study. Because chewing forces are cyclic forces. In this study, finite element analysis is limited in fully reflecting clinical conditions as it allows static forces to be measured. Therefore, this study is a preliminary study and should be confirmed by clinical studies.

There are two types of modeling; parametric surface modelling (surface first approach) and freeform mesh modelling (mesh first approach), with each having its own pros and cons in the validation of finite element models. In the surface first approach, although it has the advantages of changing the mesh detail in the later stages and the application of load and boundary conditions to the surfaces is very easy, it has disadvantages such as the difficulty of modeling complex organic shapes and the difficulty of solid lattice organic shapes in the optimum number of elements. In the mesh first approach, the modeler must estimate and decide the number of mesh needed before modeling. It is difficult to change the mesh detail size in the late stages. Also, since there are no surfaces, the user cannot select surfaces to apply boundary conditions. If the user requests to perform a surface loading operation, the user will need to select nodes as the boundary condition points. However, this approach has the advantages of making it easier to model complex geometries as freehand tools are very strong in mesh-based modelers, and in the first mesh approach, the modeler can manually adjust the level of detail to easily reach the optimum number of elements. So, we have used mesh first approach to get highly detailed and realistic organic 3d models that cannot be achieved by parametric surface modeling. The software that we have used can import the mesh models (.stl files) and perform solid modelling and analysis. By this way, we gain the advantage of working on highly realistic 3d models in the cost of losing the ability to find the convergence point. However, since our models are highly detailed and the number of meshed and nodes is far beyond any possible convergence point, we assume that we get rid of that disadvantage of the mesh first approach method.

In multiunit abutment-implant connection systems, abutment screw loosening may occur due to prosthetic loads and lead to mechanical complications such as screw fracture [23]. Ji-Hyeon et al. [24] reported that the stress in posterior implant screws was higher than that in anterior implant screws when stress distributions in prosthetic screws were examined in the all-on-four concept. In this study, the stress values in posterior implant screws were higher than those in anterior implant screws. However, the stress values observed in prosthetic screws in the B group models were higher than those in the A group models. AlHomidhi et al. [25] simulated a 5-year chewing function with a chewing simulator in a comparison study of screw-retained and multiunit screw-retained abutments. As a result, they stated that multiunit screw-retained abutments are more resistant to occlusal forces. A possible reason could be that in multiunit abutment-implant connection systems, two screws, one in the abutment-implant connection and the other between the abutment-prosthesis, reduce the occlusal forces by dividing them.

Moraes et al. [26] examined the effects in the cortical bone around dental implants of different diameters under the application of axial and oblique forces. They reported that wide-diameter implants had lower stress values in the cortical bone than regular diameter implants and axial forces compared to oblique forces. In all models of this study, stress accumulation in the cortical bone under oblique loads was higher than that under axial loads. Moreover, the highest stress value in the cortical bone under oblique loading was seen in the M1B model that had the narrowest implant diameter (3.5 mm) in group B. This finding suggests that the stress distribution depends on implant diameters. However, a comparison between both groups showed that stress values in the cortical bone were higher in group B under axial and oblique loading. In the stress assessment of the trabecular bone, the highest stress was observed in the narrowest implant in group B. Raaj et al. [27], in a comparison study of the stress values of implants of two different diameters, reported that the highest stress values were in the trabecular bone around the 3.5 mm diameter implant and the lowest in the trabecular bone around the 4.3 mm diameter implant. In this study, stress values in the trabecular bone around the implant of 3.5 mm and 4 mm diameters were found to be similar. However, in most stress values of the trabecular bone around the 4.5 mm diameter implants were found to be higher than the trabecular bone stress values around the 3.5 mm and 4 mm diameter implant. With these findings, it was concluded that the periodic increase in implant diameter is not directly proportional to the decrease in stress in the trabecular bone around the implant.

A new tilted monoblock dental implant system may be an alternative in the all-on-four concept to prevent possible infection in the multiunit abutment-implant connection. However, this system needs to be developed with further studies to reduce stress and tension values in implants, implant components, and bone. Moreover, this study should be supported by other in vitro and in vivo studies.

## Conclusions

In axial and oblique loads, Von Mises stress values in implants, cortical and trabecular bone around the implants and prosthetic screw of the implant in group B are higher than in group A.

## Data Availability

The datasets used and/or analysed during the current study are available from the corresponding author on reasonable request.

## Abbreviations

FEM: Finite element method
3-D: Three-dimension
GPa: Gigapascal
mm: Milimeter
N: Newton
GB: Gigabyte
GHz: Gigahertz
M1A: Model-1A
M2A: Model-2A
M3A: Model-3A
M1B: Model-1B
M2B: Model-2B
M3B: Model-3B
VMS: Von Mises stress
MPa: Megapascal
PS: Principal stress
